# Structural basis of ribosomal frameshifting during translation of the SARS-CoV-2 RNA genome

**DOI:** 10.1126/science.abf3546

**Published:** 2021-05-13

**Authors:** Pramod R. Bhatt, Alain Scaiola, Gary Loughran, Marc Leibundgut, Annika Kratzel, Romane Meurs, René Dreos, Kate M. O’Connor, Angus McMillan, Jeffrey W. Bode, Volker Thiel, David Gatfield, John F. Atkins, Nenad Ban

**Affiliations:** 1 Department of Biology, Institute of Molecular Biology and Biophysics, ETH Zurich, Zurich, Switzerland.; 2 School of Biochemistry and Cell Biology, University College Cork, Cork T12 XF62, Ireland.; 3 School of Microbiology, University College Cork, Cork T12 K8AF, Ireland.; 4 Institute of Virology and Immunology, University of Bern, Bern, Switzerland.; 5 Department of Infectious Diseases and Pathobiology, Vetsuisse Faculty, University of Bern, Bern, Switzerland.; 6 Graduate School for Cellular and Biomedical Sciences, University of Bern, Bern, Switzerland.; 7 Center for Integrative Genomics, Génopode, University of Lausanne, 1015 Lausanne, Switzerland.; 8 Laboratorium für Organische Chemie, Department of Chemistry and Applied Biosciences, ETH Zurich, Zurich, Switzerland.; 9 MRC Laboratory of Molecular Biology, Cambridge CB2 0QH, UK.

## Abstract

Programmed ribosomal frameshifting is a key event during translation of the severe acute respiratory syndrome coronavirus 2 (SARS-CoV-2) RNA genome that allows synthesis of the viral RNA-dependent RNA polymerase and downstream proteins. Here, we present the cryo–electron microscopy structure of a translating mammalian ribosome primed for frameshifting on the viral RNA. The viral RNA adopts a pseudoknot structure that lodges at the entry to the ribosomal messenger RNA (mRNA) channel to generate tension in the mRNA and promote frameshifting, whereas the nascent viral polyprotein forms distinct interactions with the ribosomal tunnel. Biochemical experiments validate the structural observations and reveal mechanistic and regulatory features that influence frameshifting efficiency. Finally, we compare compounds previously shown to reduce frameshifting with respect to their ability to inhibit SARS-CoV-2 replication, establishing coronavirus frameshifting as a target for antiviral intervention.

## Shifting frames to make more proteins

Severe acute respiratory syndrome coronavirus 2 critically depends on the ribosomal frameshifting that occurs between two large open reading frames in its genomic RNA for expression of viral replicase. Programmed frameshifting occurs during translation, when the ribosome encounters a stimulatory pseudoknot RNA fold. Using a combination of cryo–electron microscopy and biochemistry, Bhatt *et al.* revealed that the pseudoknot resists unfolding as it lodges at the entry of the ribosomal messenger RNA channel. This causes back slippage of the viral RNA, resulting in a minus-1 shift of the reading frame of translation. A partially folded nascent viral polyprotein forms specific interactions inside the ribosomal tunnel that can influence the efficiency of frameshifting.

*Science*, abf3546, this issue p. 1306

## Teaser

A pseudoknot impedes ribosome progression to promote frameshifting in SARS-CoV-2, which allows synthesis of the viral proteins.

Ribosomal frameshifting, a process during which the reading frame of translation is changed at the junction between open reading frames (ORFs) 1a and 1b, is one of the key events during translation of the severe acute respiratory syndrome coronavirus 2 (SARS-CoV-2) positive-sense single-stranded RNA genome. This programmed −1 translational frameshifting is conserved in all coronaviruses and is necessary for the synthesis of viral RNA-dependent RNA polymerase (RdRp or Nsp12) and downstream viral nonstructural proteins that encode core enzymatic functions involved in capping of viral RNA, RNA modification and processing, and RNA proofreading (*1*). Although the translational machinery typically prevents frameshifting as a potential source of one of the most disruptive errors in translation (*2*, *3*), many viruses rely on programmed ribosomal frameshifting to expand and fine-tune the repertoire and stoichiometry of expressed proteins (*4*).

**Fig. 1:**
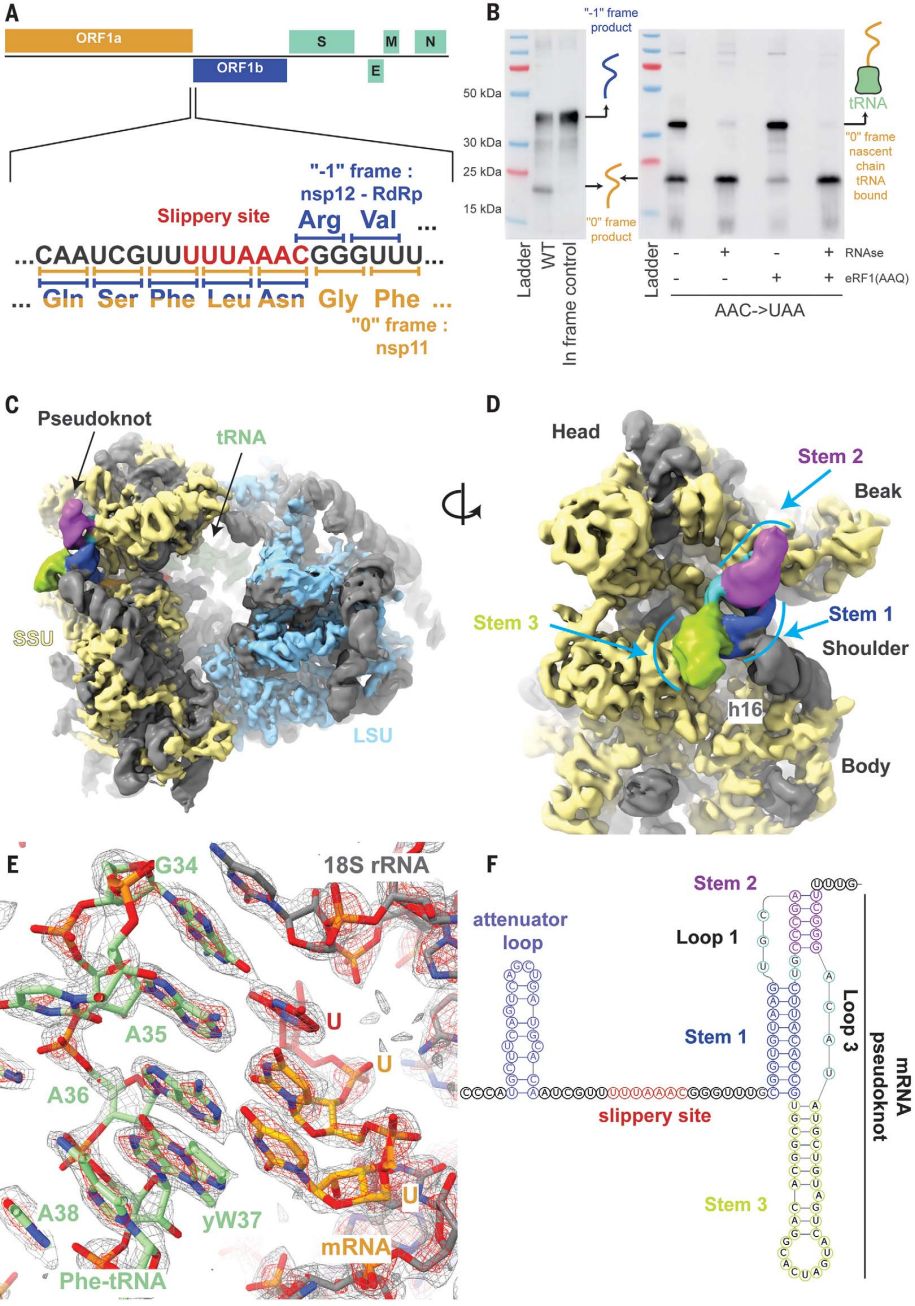
The SARS-CoV-2 pseudoknot interacts with the ribosome and pauses translation upstream of the slippery site. (**A**) Schematic of the SARS-CoV-2 main ORF. In the close-up view of the frameshift event, codons and corresponding amino acids are shown. During −1 frameshifting, the slippery site codons UUA (Leu) and AAC (Asn) are the last codons decoded in the 0 frame. Upon −1 frameshifting of the AAC codon to AAA, translation resumes at the CGG (Arg) triplet, where elongation proceeds uninterrupted to produce full-length Nsp12. (**B**) In vitro translation reaction depicting pausing at the frameshift site, as shown with Western blotting. Efficient frameshifting is observed for the WT template, consistent with our dual luciferase assays (see methods). Samples for cryo-EM originally intended to be trapped by dominant negative eRF1 (AAQ) show a tRNA-bound pause in proximity of the frameshift site. The tRNA-associated band is lost upon RNase treatment. Reactions without added eRF1 (AAQ) produce a similarly paused product. (**C**) Overview of the density low-pass filtered to 6 Å with the pseudoknot found close to the entry of the mRNA channel on the small subunit (SSU). The SSU proteins are colored in yellow, the large subunit (LSU) proteins in blue, and the rRNA in gray. The pseudoknot is colored according to its secondary structure as in (F), and the P-site tRNA is colored in green. (**D**) Close-up view of the pseudoknot from the solvent-exposed side of the SSU. Helix h16 of the 18*S* rRNA interacts with the base of Stem 1. Unpaired loop-forming nucleotides are colored in cyan. (**E**) P-site codon-anticodon interactions reveal a Phe (UUU) codon interacting with tRNA(Phe). yW37, wybutosine at position 37. (**F**) Schematic of the revised secondary structure elements in the pseudoknot necessary for −1 programmed ribosomal frameshifting, with different functional regions labeled and colored accordingly.

Programmed −1 frameshifting in SARS-related coronaviruses occurs at the slippery sequence U_UUA_AAC in the context of a 3′ stimulatory RNA sequence that was predicted to form a three-stemmed pseudoknot structure (*5*) and, in parallel, was independently tested by our lab and others (*6*–*8*). The frameshifting occurs with high efficiency (25 to 75%), depending on the system used (*6*, *7*, *9*–*11*), and changes the reading frame to UUU_AAA_C (*12*) (Fig. 1A). Consequently, two viral polyproteins are synthesized: one encoded by ORF1a when frameshifting does not take place, and ORF1ab, which is expressed as a result of frameshifting. Translation of ORF1a produces polyprotein 1a, which ends with Nsp10 followed by the short Nsp11. Conversely, when the frameshift occurs, the polyprotein 1ab is generated, which contains almost 2700 additional amino acids and in which the viral RdRp, Nsp12, is produced after Nsp10 as a consequence of translation in the −1 frame. A putative secondary structure element in the viral RNA that forms a loop upstream of the shift site has been proposed to play an attenuating role in frameshifting and is referred to as the 5′ attenuator loop (*8*). Maintaining the precise level of coronavirus frameshifting efficiency is crucial for viral infectivity, as evidenced by the fact that mutation of a single nucleotide in the frameshifting region of the SARS-CoV-1 RNA results in a concomitant abrogation of viral replication (*13*). Therefore, the importance of three-stemmed pseudoknot-dependent −1 ribosomal frameshifting for the propagation of SARS-related coronaviruses, a process that has not been seen to occur on any endogenous human transcript in human cells, presents itself as an opportune drug target with minimal tolerance for drug-resistant mutations.

Because of its importance in the life cycle of many important viruses and coronaviruses in particular, programmed frameshifting has been extensively studied using a range of structural and functional approaches (*4*). The structure of a 3′ stimulatory pseudoknot in isolation or in context of the viral genome has been proposed recently by various groups using techniques that include molecular dynamics, nuclease mapping, in vivo selective 2′-hydroxyl acylation analyzed by primer extension (SHAPE), nuclear magnetic resonance (NMR), and cryo–electron microscopy (cryo-EM) (*7*, *14*–*17*). Furthermore, a ribosomal complex with a frameshift stimulatory pseudoknot from the avian infectious bronchitis virus was reported at low resolution (*18*). Here, to provide a structural and mechanistic description of the events during ribosomal frameshifting, we investigated mammalian ribosomes captured in distinct functional states during translation of a region of SARS-CoV-2 genomic RNA where −1 programmed frameshifting occurs.

## Structure determination of a frameshifting-primed ribosomal complex

We captured a 0 frame, preframeshift ribosomal complex by introducing a stop codon in place of the second codon of the slippery site (U_UUA_AAC to U_UUA_UAA) (Fig. 1A) and adding mutant eukaryotic release factor 1 [eRF1 (AAQ)] that is unable to release the nascent polypeptide. Translating complexes were prepared in an in vitro translation reaction using an in-house–generated rabbit reticulocyte lysate (RRL) system that supported efficient frameshifting in the previously reported range of around 50% (*19*) according to dual luciferase experiments (see methods). The ribosomes were programmed with mRNA encoding an affinity tag and harboring a region of the SARS-CoV-2 genome that encodes proteins Nsp10 (C terminus), Nsp11, and most of Nsp12. Western blotting showed that when using the wild-type (WT) RNA template, frameshifting was efficient, whereas the stop codon mutation prevented frameshifting and led to ribosome pausing. This effect was further enhanced when eRF1 (AAQ) was present in excess over endogenous WT eRF1 (Fig. 1B).

**Fig. 2:**
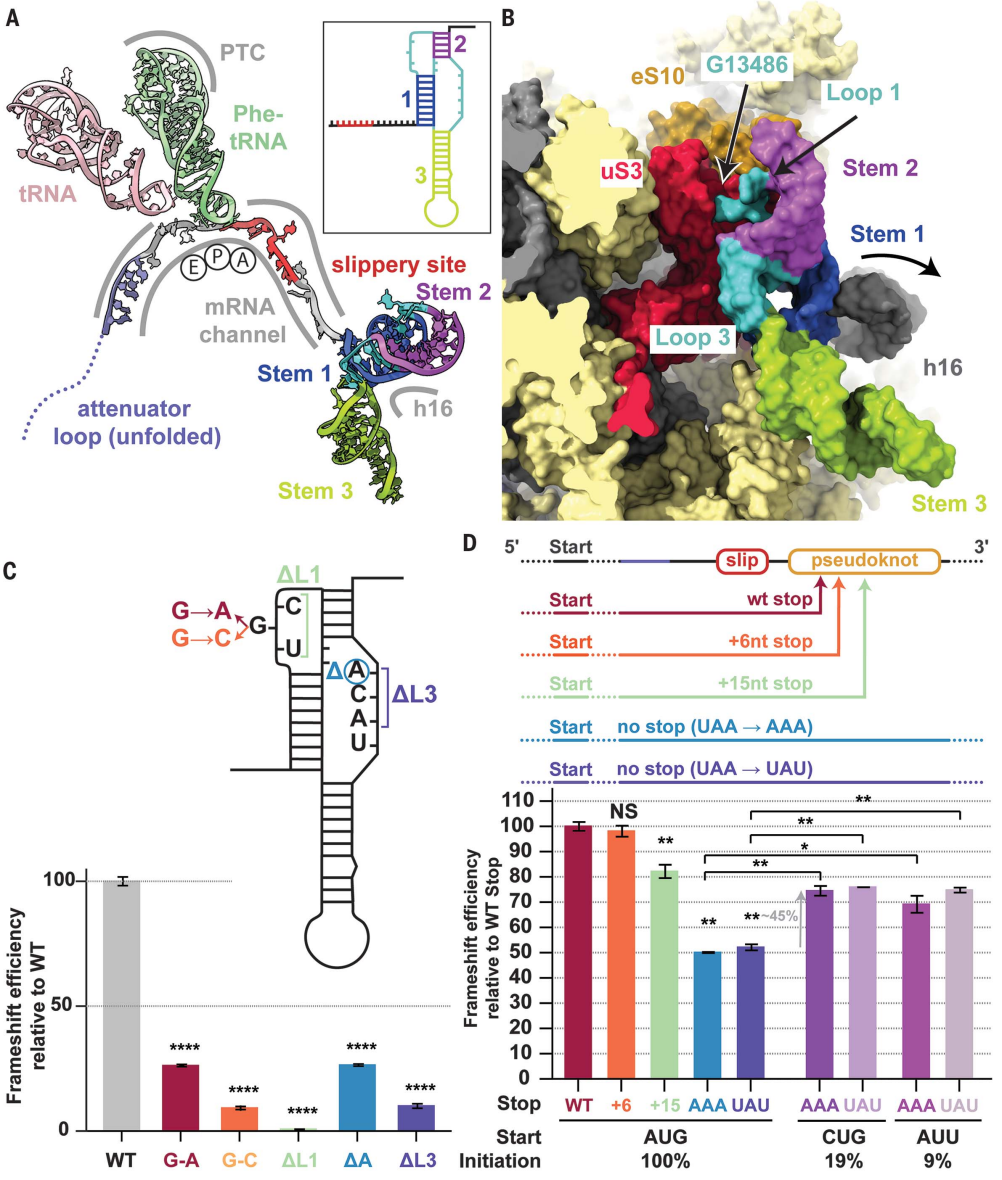
Critical features of the ribosome-bound pseudoknot. (**A**) Overview of the frameshift-primed state. The stimulatory pseudoknot pauses the ribosome at the penultimate codon (UUU) of the slippery site (red), with P-sites (green) and E-sites (pink) occupied by tRNAs and an empty A-site awaiting decoding in the nonrotated state. The length of the spacer region (gray) is critical for exact positioning of the pseudoknot as the spacer exerts tension at the entry of the mRNA channel (fig. S6C). The inset shows a secondary structure depiction of the frameshift-stimulating pseudoknot colored accordingly. PTC, peptidyl transferase center. (**B**) The backbone of Loop 1 (UGC) (cyan) of the pseudoknot interacts with the N-terminal domain of uS3 (red) and the C-terminal tail of eS10 (orange). mRNA residue G13486 is flipped out and interacts with uS3 (fig. S6D). (**C**) Mutagenesis experiments using dual luciferase assays in HEK293T cells indicate that the G13486 interaction is specific. Mutation of G13486 to other residues leads to a marked reduction in frameshifting efficiency, and deletion of Loop 1 (ΔL1) completely abolishes frameshifting. Similarly, deletion of a single nucleotide (A13537) in Loop 2 reduces frameshifting, whereas deletion of the entire loop (ΔL2) abolishes frameshifting. Normalized (Firefly-Renilla) luciferase activities were calculated for each construct as a percentage of their individual normalized in-frame controls. Data are presented as mean values ± standard deviations of three biological replicates (sets of translation reactions) averaged after three measurements, with error bars representing standard deviations. ****P < 0.0001 by Student’s two-tailed *t* test. (**D**) Mutagenesis experiments using dual luciferase reporter assays in HEK293T cells show that the position of the 0 frame stop codon influences frameshifting. When leaving the pseudoknot unaltered, an incremental increase in the distance of the 0 frame stop codon from the frameshift site leads to a concomitant decrease in frameshifting levels. Loss of the stop codon in the 0 frame leads to a sharp decline in frameshifting levels. This reduction is rescued by ~45% upon decreasing ribosome loading levels by implementing weaker initiation codons. The graph is normalized relative to the WT frameshifting of 25%. Mutations and complementary mutations are shown in fig. S8. Error bars represent standard deviation. NS, not significant; *P < 0.1; and **P < 0.01 by Student’s two-tailed *t* test.

The cryo-EM three-dimensional (3D) reconstruction of ribosome–nascent chain complexes affinity-purified from the reactions supplemented with eRF1 (AAQ) revealed two distinct ribosomal complexes captured in the process of translating the slippery sequence (figs. S1 and S2). One represented a termination complex that contained the ATP-binding cassette transporter 1 (ABCE1), which is known to be involved in termination and recycling together with mutant eRF1 interacting with the stop codon (fig. S3). The second reconstruction resolved translating 80*S* ribosomes containing bound P- and E-site tRNAs (fig. S2). This reconstruction at 2.2-Å resolution allowed us to build the most accurate structure of a mammalian 80*S* ribosome so far and directly visualize many protein and virtually all rRNA modifications identified for the human ribosome based on quantitative mass spectrometry and as interpreted in a recent human ribosome structure (*20*, *21*), consistent with the complete conservation of all modified residues between rabbit and human ribosomal RNAs (rRNAs) (figs. S4 and S5; and tables S1 to S3). Importantly, this reconstruction also featured additional density at the entrance to the mRNA channel suggestive of a structured RNA, which, after focused classification, revealed a prominent density for a complete 3′ frameshifting stimulatory pseudoknot at the entry of the mRNA channel on the 40*S* subunit (Fig. 1, C and D). The resolution of this reconstruction ranged from 2.4 Å at the core of the ribosome to ~7 Å at the periphery, where the most flexible regions of the pseudoknot are located (figs. S2 and S6). Based on the high-resolution maps that allowed visualization of the codon-anticodon interactions and modifications in the tRNA (Fig. 1E and fig. S6, A and B), we could unequivocally determine that a Phe-tRNA(Phe) was bound at the P-site (*22*). The mRNA does not adopt any unusual structure in the A-site of the ribosome as was observed for the HIV-1 frameshifting sequence visualized on the bacterial ribosome (*23*). This implied that the ribosome is paused by the downstream pseudoknot located at the entrance to the mRNA channel such that the P-site tRNA interacts with the UUU codon just prior to the first codon, UUA, of the slippery site (Fig. 2A).

## The pseudoknot causes ribosomal pausing prior to −1 frameshifting

The observation that the pseudoknot acts as an obstacle to slow down translation as the ribosome approaches the slippery site is mechanistically reasonable. Because the pseudoknot is a stable structural element in the mRNA, it will resist unfolding and consequently generate a back-pull on the viral RNA, resulting in an increased chance of −1 frameshifting as the tRNAs are translocated. A pause in translocation at a codon that precedes the slippery site, characterized by a >10 times longer occupancy prior to the slippage event, was observed in an analogous case of heptanucleotide −1 frameshifting on the bacterial *dnaX* gene using single-molecule experiments (*24*). According to this model, it would be anticipated that a further round of translocation results in unwinding of Stem 1 of the downstream stimulatory pseudoknot structure. Consistently, in our structure of the eRF1 (AAQ)–bound ribosome that advanced one codon further along the mRNA, no clear secondary structure is visible at the entrance to the mRNA channel because the mRNA now becomes disordered at this position (figs. S1 and S3, A and B).

**Fig. 3:**
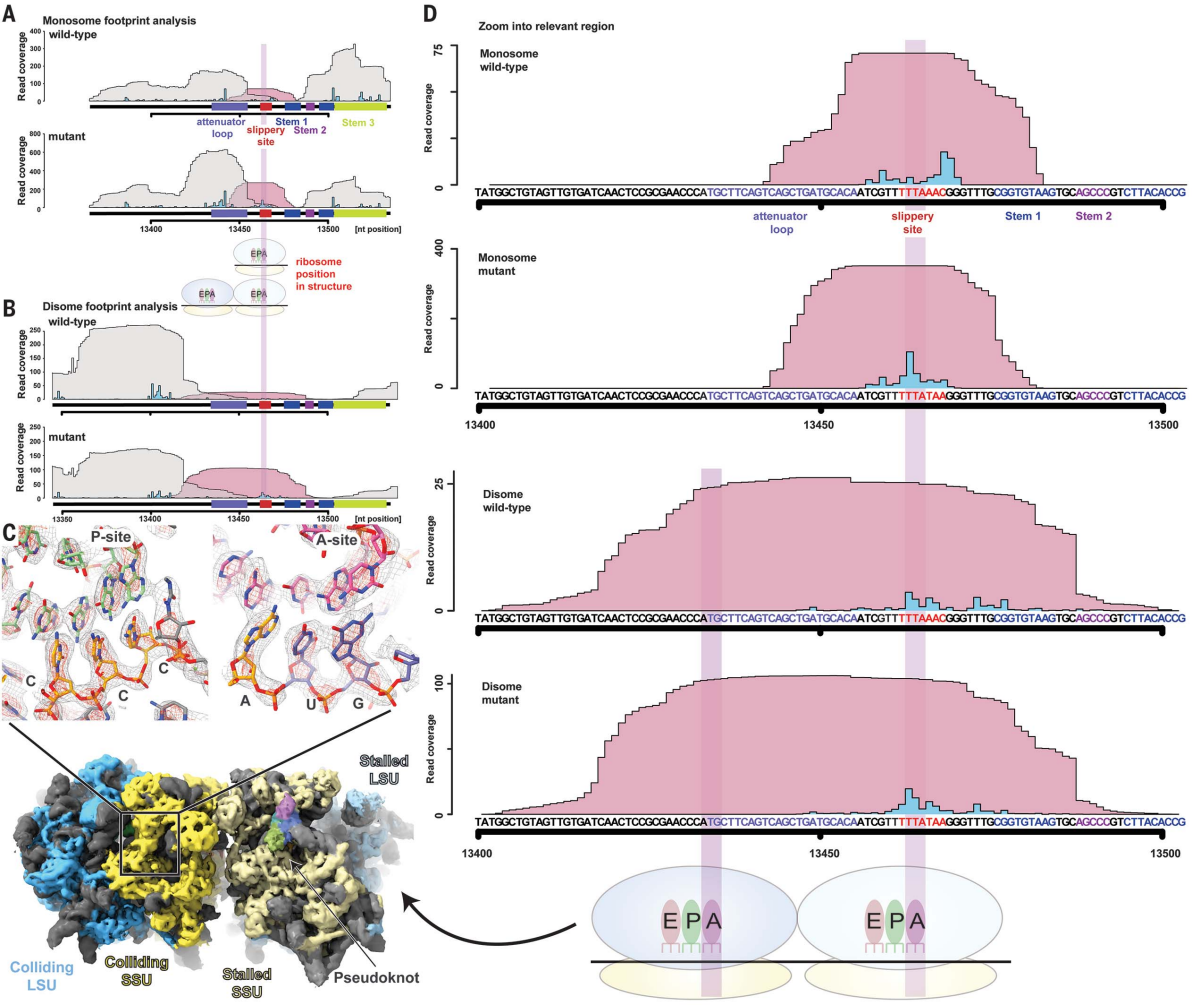
Pseudoknot-mediated pause occurs prior to the −1 frameshifting event. (**A** and **B**) Footprint coverage for WT and mutant constructs determined by monosome-selective (A) and disome-selective (B) ribosome profiling. Pileup of reads from the indicated areas are plotted separately for reads that overlap (pink) or do not overlap (gray) the frameshift site (indicated by red bar below the *x* axis). The predicted A-sites of the ribosomes that give rise to the footprints are depicted as blue peaks. A-site predictions were carried out as described in the supplementary materials. (**C**) In high-resolution cryo-EM reconstructions of disomes at the frameshift site, the P- and A-sites of the trailing ribosome show occupancy of CCC and AUG codons, respectively, corresponding to the positions estimated by disome profiling. Disome maps were calculated by separately refining the orientational parameters for each ribosome. (**D**) Magnification of the frameshift region from (A) and (B) reveals that monosome profiles show transient occupancy in the vicinity of the frameshift site, whereas disome profiles, which are indicative of strong pause sites, show a similarly enhanced occupancy at the first codon (UUA) of the frameshift site in both WT and mutant constructs. A-site codons of the leading and trailing ribosome are highlighted with a translucent bar and correspond to those seen in the disome structure in (C).

To investigate the slowdown of translation on the WT slippery sequence, we performed disome footprint profiling, a method that identifies translational pause sites through the analysis of transitory ribosome collisions (*25*–*27*) (see methods). Notably, recent studies using conventional ribosome profiling methodology reported a lack in monosome footprint coverage across the frameshifting region on the SARS-CoV-2 RNA (*11*, *28*), possibly because ribosomes in this area became trapped in temporary collisions. Moreover, the highly structured pseudoknot at the entry to the mRNA channel would likely preclude efficient trimming by ribonuclease I (RNase I), the enzyme used for footprint generation, further reducing efficient monosome footprint capture. Using a modified nuclease treatment protocol (see methods) that recovered monosome footprints from the frameshift region (Fig. 3, A and C), our experiments revealed that ribosome collisions occur as a result of ribosomal pausing at the same position that is observed in the structure of the pseudoknot-engaged ribosome (Fig. 3, B and D). Apparently, although the base substitutions creating a stop codon in the 3′ adjacent slippery site did not change the features of pausing, they increased the dwell time of the ribosomes at the pause site sufficiently to allow visualization in the cryo-EM experiment.

The results of our disome profiling experiments prompted us to structurally investigate disomes by cryo-EM. We were able to visualize the pseudoknot-paused ribosome followed by a closely trailing ribosome. Upon focused refinement, we obtained a high-resolution (3.1 Å) structure of the trailing ribosome in a rotated state (fig. S1). In congruence with our estimated positioning of the ribosomes in disome profiling (Fig. 3D), the purine-pyrimidine pattern of codon-anticodon pairs in the structure of the colliding ribosome revealed that the pause occurs with CCC and AUG triplets in the P- and A-sites, respectively (Fig. 3C).

## The SARS-CoV-2 RNA pseudoknot specifically interacts with ribosomal proteins and 18*S* rRNA

The intermediate local resolution (5 to 7 Å) of the cryo-EM map in the area of the pseudoknot allowed us to visualize the overall fold of the RNA and readjust its previously predicted secondary structure (*14*–*17*, *19*) (Fig. 1, C, D, and F). The stimulatory pseudoknot forms an H-type pseudoknot with Stem 1 and Stem 2 coaxially stacked on top of each other to form a quasi-continuous helix, whereas Stem 3 stands out almost perpendicular to this plane (Figs. 1D and 2B). This corkscrew-like formation provides a bulky and well-structured obstacle wedged at the mRNA entry channel, which has the potential to resist unwinding by the helicase activity of the ribosome and generate tension on the upstream mRNA up to the decoding center. Stem 1 of the pseudoknot forms a 9–base pair helix that is GC rich at the bottom (Fig. 1F). The penultimate nucleotides of the “spacer region” before Stem 1 are located at the mRNA entry tunnel, where they interact with several basic residues in the C-terminal domain of uS3 on one side and are supported by uS5 from the other, with an additional weak contact contributed by the C-terminal end of eS30. uS3 and eS30 are primary components of the ribosome helicase, and uS5 has been proposed to be a component of the ribosomal helicase processivity clamp at the mRNA entry site (*29*, *30*). The observed distance between the P-site UUU codon and Stem 1 of the pseudoknot underscores the critical dependence of the frameshifting efficiency on the length of the spacer region (*31*). Translocation to the next codon would place the frameshifting codon UUA into the P-site, with a simultaneous increase in the tension of the mRNA and unwinding of the GC-rich base of Stem 1 upon entering the mRNA entry channel, comparable to the situation when the ribosome proceeds to the engineered stop codon, as observed in our eRF1 (AAQ)–stalled structure (fig. S3).

The pseudoknot structure also reveals a hitherto unobserved and possibly unappreciated role for the distal site of the mRNA entrance channel in helicase activity. Although mRNA unwinding studies outside the mRNA entrance channel have so far implicated only a helix in the C-terminal domain of uS3 (*32*), we noticed that Loop 1 of the pseudoknot contacts the N-terminal domain of uS3 as well as the C-terminal tail of eS10 (Fig. 2B and fig. S6D), whereas the flipped-out base G13486 in this loop forms specific interactions (Fig. 2B). Furthermore, because the pseudoknot is located at the entry to the mRNA channel, helix h16 of the 18*S* rRNA is noticeably pushed outward owing to a direct contact with the minor groove of Stem 1 (Fig. 2B and fig. S7A). Because the pseudoknot wedges between the head and the body of the small ribosomal subunit, it would restrict their relative motions that need to take place during translocation. This is consistent with the studies on dynamics of coronavirus frameshifting, which revealed that the mechanism of −1 frameshifting involves restriction of small subunit head motion (*33*).

The structure also reveals another key aspect of the architecture of the pseudoknot as the ribosome encounters it. The start of the pseudoknot is shifted relative to the predicted secondary structure (*14*–*17*, *19*) by two nucleotides. The two opposed nucleotides, which were assumed to base pair with Stem 1, are actually forming the start of Stem 3 by pairing with bases predicted to be in the single-stranded linker 2 (Fig. 1F and fig. S7, B and C). Our cryo-EM density reveals that Loop 3 accommodates a total of four nucleotides, three of which were originally attributed to Stem 2. Thus, we observe that Loop 3 is shifted and expanded relative to the initially predicted secondary structures (*14*–*17*, *19*).

To functionally support our structural findings and confirm the nature and specificity of the pseudoknot interactions, we performed structure-guided mutagenesis experiments using dual luciferase reporter assays in human embryonic kidney (HEK) 293T cells (see methods) and monitored the frameshifting efficiency relative to the WT (Fig. 2C). Mutation of G13486 of Loop 1 to another purine reduced the frameshifting efficiency to 30% of the WT level, and mutation of this base to a pyrimidine further reduced frameshifting to 15%. As expected from our structural data, deletions of the nucleotides of the spacer regions also had a deteriorating effect on frameshifting. Loss of Loop 1 entirely abolished frameshifting. Deletion of a single nucleotide of Loop 3 in agreement with its proposed role in forming the base-pairing interactions diminished the frameshifting rate to 25% of the WT level. Loss of the entire Loop 3 reduced frameshifting to 10% of WT levels.

## Frameshifting efficiency depends on the position of the 0 frame stop codon

In SARS-CoV-2, the 0 frame stop codon is located five codons downstream of the frameshift site and is a constituent of Stem 1. The placement of the stop codon in such proximity to the frameshift site is a common feature in coronaviruses, and its presence in a critical region of the stimulatory pseudoknot prompted us to probe the effect of the distance of the 0 frame stop codon on frameshifting. To this end, knowledge of the 3D structure of the pseudoknot helped us to confidently manipulate the stop codon without hampering pseudoknot formation. We introduced mutations to incrementally extend the stop codon from the WT position and to completely remove the occurrence of a stop codon in the 0 frame (Fig. 2D and fig. S8). Whereas introducing a stop codon six nucleotides downstream of the WT position only marginally decreased the frameshifting rate (98% of WT), a stronger attenuation was observed when the distance of the stop codon was increased to 15 nucleotides from the WT stop (80% of WT). Finally, removal of the stop codon by two different point mutations led to a reduction of frameshifting efficiency to 50% of WT levels. To test whether reduced ribosomal loading rescues the effect of stop codon removal, we analyzed the frameshifting efficiency in the context of weaker initiation codons such as CUG and AUU (Fig. 2D). These constructs led to a 45% rescue of the reduction in frameshifting compared with stop codon mutants initiating at an AUG start.

Taken together, these observations suggest that the stop codon position plays an important role in maintaining optimum frameshift efficiency. We propose that the stop codon serves to prevent the closely trailing ribosome from encountering a viral RNA that was unfolded by the leading ribosome. In this case, upon encountering a stop codon, termination and subunit disassembly will occur, which will provide an opportunity for the pseudoknot to refold without the constraints of the mRNA channel (see Conclusions). According to this model, although the WT stop codon will make the frameshifting efficiency less sensitive to ribosome loading in the “no-frameshifting” scenario, the frameshifting events that occur after a −1 frameshift will nevertheless be more likely when the ribosomes are spaced further apart. Our measurements of the efficiency of frameshifting for the WT sequence in the context of different rates of translation initiation are in agreement with this hypothesis (fig. S9). This mechanism, consistent with our biochemical data, increases the efficiency of frameshifting to the levels required by SARS-CoV-2 and may be used by viruses in general when high-efficiency frameshifting is required.

## Nascent chain forms specific interactions with the ribosomal exit tunnel

**Fig. 4:**
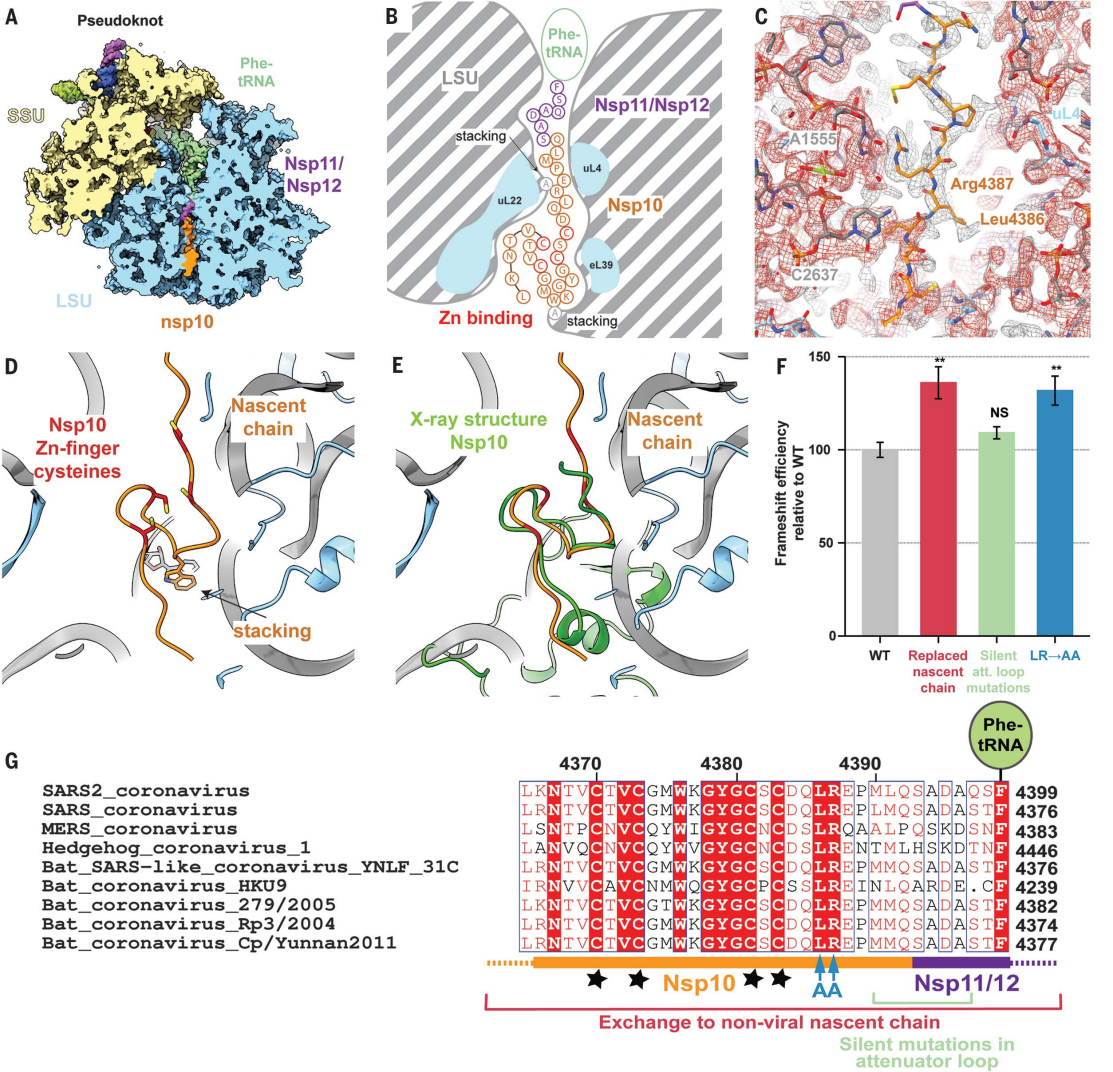
The nascent viral polypeptide cotranslationally folds and specifically interacts with the ribosomal tunnel. (**A**) Cross-section of the pseudoknot-paused ribosome structure showing the exit tunnel. The nascent C terminus of Nsp10 (orange) and the N terminus of Nsp11/Nsp12 (purple) are visible from the PTC to the periphery of the ribosome exit tunnel (LSU in blue). (**B**) Schematic representation of the path of nascent peptide along the exit tunnel. Arg^4387^ stacks with 28*S* rRNA residue A^1555^ at the constriction site. Further down, where the tunnel widens, the C-terminal zinc finger domain of Nsp10 folds cotranslationally, with Trp^4376^ stacking on A^2261^ of 28*S* rRNA. (**C**) A well-ordered density is visible for Arg^4387^ of Nsp10 as it stacks onto A^1555^ of 28*S* rRNA at the constriction site and is stabilized by Leu^4386^. The structure is shown within the cryo-EM map contoured at two different levels (gray and red). (**D** and **E**) The overlay of the cotranslationally folded zinc finger domain with the crystal structure of Nsp10 [green, PDB 2FYG (*37*)] reveals the structural similarity. (**F**) Probing the role of nascent chain interactions with the ribosome exit tunnel using an RRL in vitro system. Mutations of the interacting residues were tested for their effect on frameshifting shown in comparison to the WT frameshifting (41% frameshifting was normalized to 100%). Replacement of the entire nascent chain with an unrelated sequence leads to a 35% relative increase in frameshifting, which is only in part due to the loss of the 5′ attenuator loop. Interactions around the constriction site likely serve to attenuate frameshifting, because replacement of the interacting Arg^4387^ and stabilizing Leu^4386^ (LR) with Ala (AA) increases frameshifting by 30%. Error bars represent standard deviation. NS, not significant, and **P < 0.01 by Student’s two-tailed *t* test. (**G**) Alignment of SARS2 with closely related sequences of other coronaviruses highlighting the conservation of the mutated residues [colored as in (F)]. The shown sequence stretch encompasses the C-terminal zinc finger domain of Nsp10 (orange) and parts of Nsp11/Nsp12 (purple) visible in our reconstruction. Nascent-chain residues Leu^4386^ and Arg^4387^ that interact with the ribosomal exit tunnel are strictly conserved, whereas the conservation of neighboring residues is lower. Stars represent the four cysteines of the Nsp10 zinc finger. Single-letter abbreviations for the amino acid residues are as follows: A, Ala; C, Cys; D, Asp; E, Glu; F, Phe; G, Gly; H, His; I, Ile; K, Lys; L, Leu; M, Met; N, Asn; P, Pro; Q, Gln; R, Arg; S, Ser; T, Thr; V, Val; W, Trp; and Y, Tyr.

Notably, in the reconstruction of the paused translating ribosome, the nascent chain that corresponds to the viral polyprotein was visible along the entire length of the ribosomal exit tunnel (Fig. 4A). The density corresponds to the C-terminal region of Nsp10, which is the activator of the viral proofreading exonuclease and N7-methyltransferase Nsp14 (*34*, *35*), and then (depending on the frameshifting event) continues as either the viral RNA-dependent RNA polymerase Nsp12 (*6*) or as protein Nsp11, whose function is still unknown (Figs. 1A and 4B). The nascent chain makes several specific interactions with the ribosomal tunnel, one of which is at the constriction site where Arg^4387^ of Nsp10 interacts with A^1555^ of the 28*S* rRNA [corresponding to A^1600^ in humans, numbering according to PDB 6EK0 (*36*)] and is stabilized by the preceding Leu^4386^ (Fig. 4C). Notably, these two amino acids are highly conserved across multiple coronaviruses (Fig. 4G), although they are located in the unstructured C-terminal region of Nsp10 and therefore considered not to be important for the fold of the protein (*37*).

Further down the tunnel, the C-terminal end of Nsp10 adopts a partially folded zinc finger motif (Fig. 4, D and E), which, upon superposition, reveals similarity with the corresponding fully folded C-terminal domain previously observed in the crystal structure of SARS-CoV-1 Nsp10 (*37*). Trp^4376^, which is located between the two pairs of cysteines that form the zinc finger, stacks with A^2261^ (A^2418^), an interaction that might serve to promote the change of nascent chain direction and facilitate folding of the zinc finger at the end of the exit tunnel. Cotranslational events, such as insertion of a transmembrane domain at the exit of the ribosomal tunnel, were shown to promote −1 ribosomal frameshifting in alphaviruses (*38*).

To investigate whether the observed contacts between the nascent chain and the ribosomal tunnel are specific and whether these interactions and cotranslational folding of Nsp10 might play a role in modulating the frameshifting process, we used our dual luciferase reporter assay to measure the frameshifting efficiency of WT and mutant nascent chain sequence constructs. Because our measurements in HEK293T cells did not reveal an appreciable change of frameshift efficiency, we carried out the same experiments in vitro using RRL to monitor the effects in a single mRNA setup. Replacement of the entire nascent chain with an unrelated sequence leads to a 35% increase in frameshifting (Fig. 4F). Importantly, this effect was provoked by the change in peptide sequence and not simply by the loss of the 5′ attenuator loop, given that a reporter containing silent attenuator loop mutations resulted in only a slight increase in frameshifting (Fig. 4F). Mutation of the Leu^4386^ and Arg^4387^ to alanine led to a considerable (30%) increase in frameshifting (Fig. 4, F and G), implying that these nascent chain interactions with the ribosomal exit tunnel play an important role in regulating frameshifting levels, possibly mechanistically akin to the well-studied SecM stalling system in bacteria (*39*), where it was shown that cotranslational folding and the translocon-induced mechanical force can rescue the stall induced by interactions between the nascent chain and the ribosomal tunnel (*40*). These observations also suggest that any cellular nascent chain factors (*41*, *42*) might influence the rate of frameshifting.

## Inhibition of viral replication by a compound that targets the SARS-CoV-2 pseudoknot

The sensitivity of the coronavirus to the finely controlled frameshifting levels (*13*) may present an opportunity to develop compounds that interfere with the frameshifting process and thus inhibit replication of the virus. Using computational modeling and reporter assays, compounds that have been predicted to bind the pseudoknot and inhibit SARS-CoV-2 frameshifting were described (*19*, *43*) but never tested with respect to their ability to inhibit viral replication. Furthermore, the fluoroquinolone compound merafloxacin was recently reported to also inhibit −1 frameshifting efficiency of SARS-CoV-2 and other betacoronaviruses (*44*). To demonstrate that the inhibition of frameshifting is a plausible strategy for drug development, we compared two of the previously described compounds with respect to their ability to reduce viral levels in infected African green monkey VeroE6 cells (fig. S10 and methods). Our experiments demonstrate that merafloxacin is a better candidate compound because it showed a concentration-dependent inhibition of frameshifting, whereas, contrary to earlier reports (*19*, *43*), the small-molecule ligand MTDB did not specifically inhibit frameshifting under our experimental conditions (fig. S10). The two compounds showed no cellular toxicity and resulted in a three to four orders of magnitude reduction of SARS-CoV-2 titer, with a half-maximal inhibitory concentration (IC_50_) of 48 μΜ for MTDB and an order of magnitude higher efficacy for merafloxacin, with an IC_50_ of 4.3 μΜ (fig. S10). Because MTDB did not appear to affect frameshifting in our reporter construct experiments in vitro and in vivo, it is possible that it inhibits SARS-CoV-2 replication by a different mechanism. Although the potency range for these compounds is not what would be expected from potential drug candidates, it nevertheless provides a starting point for high-throughput screening and establishes that frameshifting is a viable target for therapeutic intervention against SARS-CoV-2.

## Conclusions

**Fig. 5:**
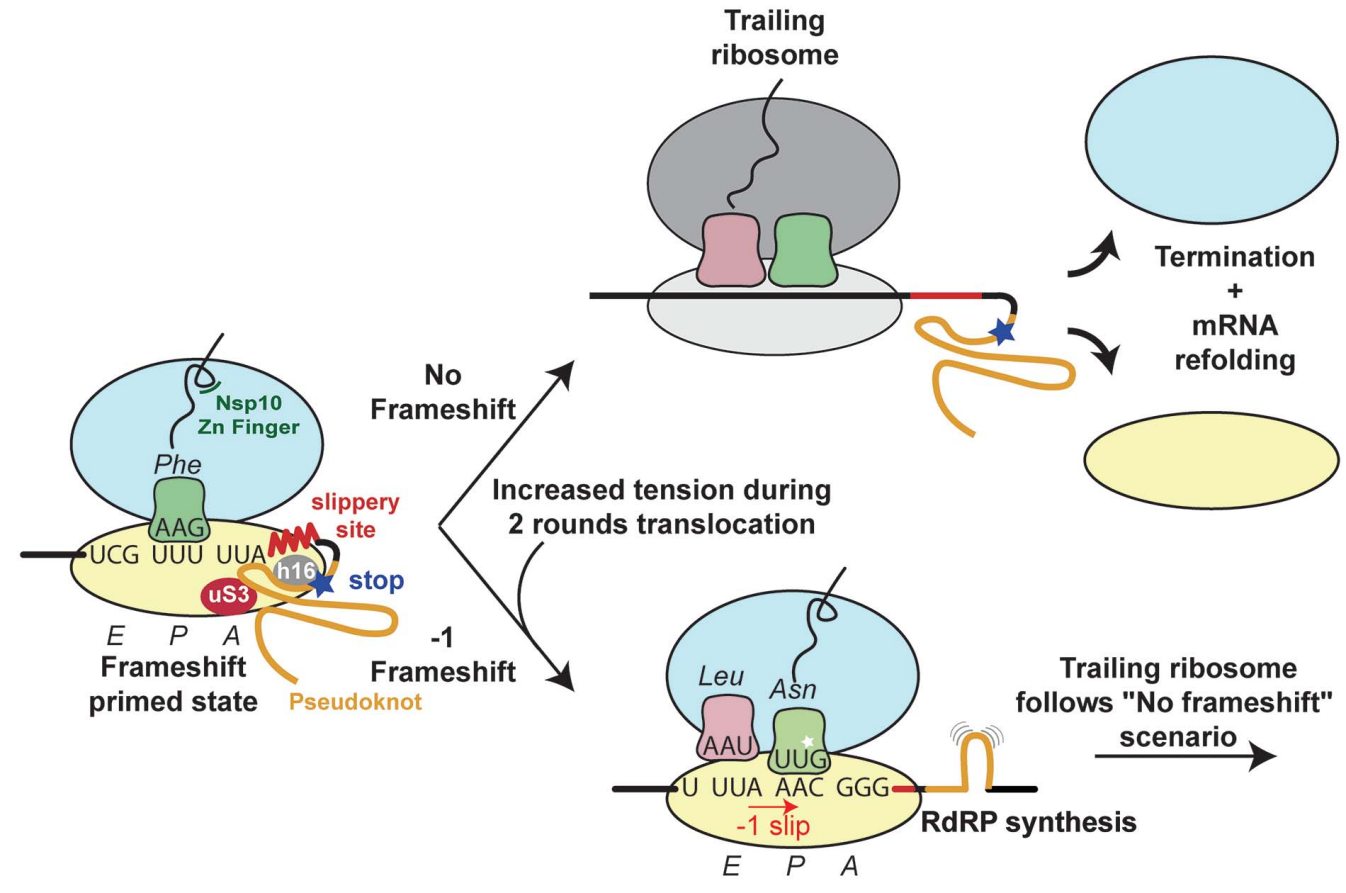
Structure-based model for −1 programmed frameshifting in coronaviruses and its regulation. The observed interactions between the pseudoknot and the ribosome prime the system for frameshifting. The features of the pseudoknot and the interactions between the nascent chain and the ribosomal tunnel play a role in the efficiency of frameshifting. The efficiency of frameshifting is increased by the presence of a stop codon near the frameshifting site. Ribosomes that progress beyond the frameshifting site in the 0 frame quickly terminate and disassemble, thereby increasing the chances that the pseudoknot will refold before it is encountered by the closely trailing ribosome. The trailing ribosome in turn encounters the pseudoknot, which increases the possibility of undergoing −1 frameshifting.

Our results provide a mechanistic description of frameshifting that occurs during translation of the SARS-CoV-2 genome and reveal the features that may be exploited by the virus to finely control the stoichiometry of viral proteins at different stages of infection (Fig. 5). Interfering with the frameshifting process at the level of nascent chain interactions with the ribosomal tunnel, at the level of RNA folding that leads to the formation of the frameshift stimulatory pseudoknot, or to perturb the interactions between the pseudoknot and the mRNA channel represent viable strategies in our search for new drugs against SARS-CoV-2, the virus that is currently causing the global COVID-19 pandemic. Our results will also be useful for understanding the mechanism of programmed ribosomal −1 frameshifting (*4*), including that used by many other medically important viruses.

## Supplementary Materials

science.sciencemag.org/content/372/6548/1306/suppl/DC1
Materials and Methods Figs. S1 to S11 Tables S1 to S4 References (*44*–*78*) MDAR Reproducibility Checklist

## Appendix

View/request a protocol for this paper from *Bio-protocol*.

## References

[1] P. V’kovski, A. Kratzel, S. Steiner, H. Stalder, V. Thiel, Coronavirus biology and replication: Implications for SARS-CoV-2. Nat. Rev. Microbiol. 19, 155–170 (2021). 10.1038/s41579-020-00468-6 33116300 PMC7592455

[2] J. Parker, Errors and alternatives in reading the universal genetic code. Microbiol. Rev. 53, 273–298 (1989). 10.1128/MR.53.3.273-298.1989 2677635 PMC372737

[3] J. M. Ogle, A. P. Carter, V. Ramakrishnan, Insights into the decoding mechanism from recent ribosome structures. Trends Biochem. Sci. 28, 259–266 (2003). 10.1016/S0968-0004(03)00066-5 12765838

[4] J. F. Atkins, G. Loughran, P. R. Bhatt, A. E. Firth, P. V. Baranov, Ribosomal frameshifting and transcriptional slippage: From genetic steganography and cryptography to adventitious use. Nucleic Acids Res. 44, 7007–7078 (2016). 10.1093/nar/gkw530 27436286 PMC5009743

[5] F. Dos Ramos, M. Carrasco, T. Doyle, I. Brierley, Programmed -1 ribosomal frameshifting in the SARS coronavirus. Biochem. Soc. Trans. 32, 1081–1083 (2004). 10.1042/BST0321081 15506971

[6] P. V. Baranov, C. M. Henderson, C. B. Anderson, R. F. Gesteland, J. F. Atkins, M. T. Howard, Programmed ribosomal frameshifting in decoding the SARS-CoV genome. Virology 332, 498–510 (2005). 10.1016/j.virol.2004.11.038 15680415 PMC7111862

[7] E. P. Plant, G. C. Pérez-Alvarado, J. L. Jacobs, B. Mukhopadhyay, M. Hennig, J. D. Dinman, A three-stemmed mRNA pseudoknot in the SARS coronavirus frameshift signal. PLOS Biol. 3, e172 (2005). 10.1371/journal.pbio.0030172 15884978 PMC1110908

[8] M. C. Su, C. T. Chang, C. H. Chu, C. H. Tsai, K. Y. Chang, An atypical RNA pseudoknot stimulator and an upstream attenuation signal for -1 ribosomal frameshifting of SARS coronavirus. Nucleic Acids Res. 33, 4265–4275 (2005). 10.1093/nar/gki731 16055920 PMC1182165

[9] I. Brierley, P. Digard, S. C. Inglis, Characterization of an efficient coronavirus ribosomal frameshifting signal: Requirement for an RNA pseudoknot. Cell 57, 537–547 (1989). 10.1016/0092-8674(89)90124-4 2720781 PMC7133225

[10] N. Irigoyen, A. E. Firth, J. D. Jones, B. Y. W. Chung, S. G. Siddell, I. Brierley, High-resolution analysis of coronavirus gene expression by RNA sequencing and ribosome profiling. PLOS Pathog. 12, e1005473 (2016). 10.1371/journal.ppat.1005473 26919232 PMC4769073

[11] Y. Finkel, O. Mizrahi, A. Nachshon, S. Weingarten-Gabbay, D. Morgenstern, Y. Yahalom-Ronen, H. Tamir, H. Achdout, D. Stein, O. Israeli, A. Beth-Din, S. Melamed, S. Weiss, T. Israely, N. Paran, M. Schwartz, N. Stern-Ginossar, The coding capacity of SARS-CoV-2. Nature 589, 125–130 (2021). 10.1038/s41586-020-2739-1 32906143

[12] V. Thiel, K. A. Ivanov, Á. Putics, T. Hertzig, B. Schelle, S. Bayer, B. Weißbrich, E. J. Snijder, H. Rabenau, H. W. Doerr, A. E. Gorbalenya, J. Ziebuhr, Mechanisms and enzymes involved in SARS coronavirus genome expression. J. Gen. Virol. 84, 2305–2315 (2003). 10.1099/vir.0.19424-0 12917450

[13] E. P. Plant, R. Rakauskaite, D. R. Taylor, J. D. Dinman, Achieving a golden mean: Mechanisms by which coronaviruses ensure synthesis of the correct stoichiometric ratios of viral proteins. J. Virol. 84, 4330–4340 (2010). 10.1128/JVI.02480-09 20164235 PMC2863758

[14] R. Rangan, I. N. Zheludev, R. J. Hagey, E. A. Pham, H. K. Wayment-Steele, J. S. Glenn, R. Das, RNA genome conservation and secondary structure in SARS-CoV-2 and SARS-related viruses: A first look. RNA 26, 937–959 (2020). 10.1261/rna.076141.120 32398273 PMC7373990

[15] K. Zhang, I. N. Zheludev, R. J. Hagey, M. T. Wu, R. Haslecker, Y. J. Hou, R. Kretsch, G. D. Pintilie, R. Rangan, W. Kladwang, S. Li, E. A. Pham, C. Bernardin-Souibgui, R. S. Baric, T. P. Sheahan, V. D Souza, J. S. Glenn, W. Chiu, R. Das, Cryo-electron microscopy and exploratory antisense targeting of the 28-kDa frameshift stimulation element from the SARS-CoV-2 RNA genome. bioRxiv 2020.07.18.209270 [Preprint]. 20 July 2020; 10.1101/2020.07.18.209270.

[16] T. C. T. Lan, M. F. Allan, L. E. Malsick, S. Khandwala, S. Y. Nyeo, Y. Sun, J. U. Guo, M. Bathe, A. Griffiths, S. Rouskin, Insights into the secondary structural ensembles of the full SARS-CoV-2 RNA genome in infected cells. bioRxiv 2020.06.29.178343 [Preprint]. 19 February 2021; 10.1101/2020.06.29.178343.

[17] N. C. Huston, H. Wan, M. S. Strine, R. de Cesaris Araujo Tavares, C. B. Wilen, A. M. Pyle, Comprehensive in vivo secondary structure of the SARS-CoV-2 genome reveals novel regulatory motifs and mechanisms. Mol. Cell 81, 584–598.e5 (2021). 10.1016/j.molcel.2020.12.041 33444546 PMC7775661

[18] O. Namy, S. J. Moran, D. I. Stuart, R. J. C. Gilbert, I. Brierley, A mechanical explanation of RNA pseudoknot function in programmed ribosomal frameshifting. Nature 441, 244–247 (2006). 10.1038/nature04735 16688178 PMC7094908

[19] J. A. Kelly, A. N. Olson, K. Neupane, S. Munshi, J. San Emeterio, L. Pollack, M. T. Woodside, J. D. Dinman, Structural and functional conservation of the programmed -1 ribosomal frameshift signal of SARS coronavirus 2 (SARS-CoV-2). J. Biol. Chem. 295, 10741–10748 (2020). 10.1074/jbc.AC120.013449 32571880 PMC7397099

[20] M. Taoka, Y. Nobe, Y. Yamaki, K. Sato, H. Ishikawa, K. Izumikawa, Y. Yamauchi, K. Hirota, H. Nakayama, N. Takahashi, T. Isobe, Landscape of the complete RNA chemical modifications in the human 80S ribosome. Nucleic Acids Res. 46, 9289–9298 (2018). 10.1093/nar/gky811 30202881 PMC6182160

[21] W. Li, S. T. L. Chang, F. R. Ward, J. H. D. Cate, Selective inhibition of human translation termination by a drug-like compound. Nat. Commun. 11, 4941 (2020). 10.1038/s41467-020-18765-2 33009412 PMC7532171

[22] G. Keith, G. Dirheimer, The primary structure of rabbit, calf and bovine liver tRNAPhe. *Biochim. Biophys. Acta* 517, 133–149 (1978). 10.1016/0005-2787(78)90041-2 414781

[23] C. Bao, S. Loerch, C. Ling, A. A. Korostelev, N. Grigorieff, D. N. Ermolenko, mRNA stem-loops can pause the ribosome by hindering A-site tRNA binding. eLife 9, e55799 (2020). 10.7554/eLife.55799 32427100 PMC7282821

[24] J. Choi, S. O’Loughlin, J. F. Atkins, J. D. Puglisi, The energy landscape of -1 ribosomal frameshifting. Sci. Adv. 6, eaax6969 (2020). 10.1126/sciadv.aax6969 31911945 PMC6938710

[25] P. Han, Y. Shichino, T. Schneider-Poetsch, M. Mito, S. Hashimoto, T. Udagawa, K. Kohno, M. Yoshida, Y. Mishima, T. Inada, S. Iwasaki, Genome-wide survey of ribosome collision. Cell Rep. 31, 107610 (2020). 10.1016/j.celrep.2020.107610 32375038 PMC7746506

[26] A. B. Arpat, A. Liechti, M. De Matos, R. Dreos, P. Janich, D. Gatfield, Transcriptome-wide sites of collided ribosomes reveal principles of translational pausing. Genome Res. 30, 985–999 (2020). 10.1101/gr.257741.119 32703885 PMC7397865

[27] S. Meydan, N. R. Guydosh, Disome and trisome profiling reveal genome-wide targets of ribosome quality control. Mol. Cell 79, 588–602.e6 (2020). 10.1016/j.molcel.2020.06.010 32615089 PMC7484464

[28] M. Puray-Chavez, K. Tenneti, H. R. Vuong, N. Lee, Y. Liu, A. Horani, T. Huang, J. B. Case, W. Yang, M. S. Diamond, S. L. Brody, J. Dougherty, S. B. Kutluay, S. B. Kutluay, The translational landscape of SARS-CoV-2 and infected cells. bioRxiv 2020.11.03.367516 [Preprint]. 16 November 2020; 10.1101/2020.11.03.367516. 10.1101/2020.11.03.367516

[29] J. Rabl, M. Leibundgut, S. F. Ataide, A. Haag, N. Ban, Crystal structure of the eukaryotic 40S ribosomal subunit in complex with initiation factor 1. Science 331, 730–736 (2011). 10.1126/science.1198308 21205638

[30] S. Takyar, R. P. Hickerson, H. F. Noller, mRNA helicase activity of the ribosome. Cell 120, 49–58 (2005). 10.1016/j.cell.2004.11.042 15652481

[31] Z. Lin, R. J. C. Gilbert, I. Brierley, Spacer-length dependence of programmed -1 or -2 ribosomal frameshifting on a U6A heptamer supports a role for messenger RNA (mRNA) tension in frameshifting. Nucleic Acids Res. 40, 8674–8689 (2012). 10.1093/nar/gks629 22743270 PMC3458567

[32] H. Amiri, H. F. Noller, Structural evidence for product stabilization by the ribosomal mRNA helicase. RNA 25, 364–375 (2019). 10.1261/rna.068965.118 30552154 PMC6380275

[33] N. Caliskan, V. I. Katunin, R. Belardinelli, F. Peske, M. V. Rodnina, Programmed -1 frameshifting by kinetic partitioning during impeded translocation. Cell 157, 1619–1631 (2014). 10.1016/j.cell.2014.04.041 24949973 PMC7112342

[34] M. Bouvet, A. Lugari, C. C. Posthuma, J. C. Zevenhoven, S. Bernard, S. Betzi, I. Imbert, B. Canard, J. C. Guillemot, P. Lécine, S. Pfefferle, C. Drosten, E. J. Snijder, E. Decroly, X. Morelli, Coronavirus Nsp10, a critical co-factor for activation of multiple replicative enzymes. J. Biol. Chem. 289, 25783–25796 (2014). 10.1074/jbc.M114.577353 25074927 PMC4162180

[35] E. C. Smith, J. B. Case, H. Blanc, O. Isakov, N. Shomron, M. Vignuzzi, M. R. Denison, Mutations in coronavirus nonstructural protein 10 decrease virus replication fidelity. J. Virol. 89, 6418–6426 (2015). 10.1128/JVI.00110-15 25855750 PMC4474304

[36] S. K. Natchiar, A. G. Myasnikov, H. Kratzat, I. Hazemann, B. P. Klaholz, Visualization of chemical modifications in the human 80S ribosome structure. Nature 551, 472–477 (2017). 10.1038/nature24482 29143818

[37] J. S. Joseph, K. S. Saikatendu, V. Subramanian, B. W. Neuman, A. Brooun, M. Griffith, K. Moy, M. K. Yadav, J. Velasquez, M. J. Buchmeier, R. C. Stevens, P. Kuhn, Crystal structure of nonstructural protein 10 from the severe acute respiratory syndrome coronavirus reveals a novel fold with two zinc-binding motifs. J. Virol. 80, 7894–7901 (2006). 10.1128/JVI.00467-06 16873246 PMC1563791

[38] H. R. Harrington, M. H. Zimmer, L. M. Chamness, V. Nash, W. D. Penn, T. F. Miller III, S. Mukhopadhyay, J. P. Schlebach, Cotranslational folding stimulates programmed ribosomal frameshifting in the alphavirus structural polyprotein. J. Biol. Chem. 295, 6798–6808 (2020). 10.1074/jbc.RA120.012706 32169904 PMC7242702

[39] H. Nakatogawa, K. Ito, The ribosomal exit tunnel functions as a discriminating gate. Cell 108, 629–636 (2002). 10.1016/S0092-8674(02)00649-9 11893334

[40] D. H. Goldman, C. M. Kaiser, A. Milin, M. Righini, I. Tinoco Jr.., C. Bustamante, Ribosome. Mechanical force releases nascent chain-mediated ribosome arrest in vitro and in vivo. Science 348, 457–460 (2015). 10.1126/science.1261909 25908824 PMC4618485

[41] G. Kramer, D. Boehringer, N. Ban, B. Bukau, The ribosome as a platform for co-translational processing, folding and targeting of newly synthesized proteins. Nat. Struct. Mol. Biol. 16, 589–597 (2009). 10.1038/nsmb.1614 19491936

[42] K. Döring, N. Ahmed, T. Riemer, H. G. Suresh, Y. Vainshtein, M. Habich, J. Riemer, M. P. Mayer, E. P. O’Brien, G. Kramer, B. Bukau, Profiling Ssb-nascent chain interactions reveals principles of Hsp70-assisted folding. Cell 170, 298–311.e20 (2017). 10.1016/j.cell.2017.06.038 28708998 PMC7343536

[43] S. J. Park, Y. G. Kim, H. J. Park, Identification of RNA pseudoknot-binding ligand that inhibits the -1 ribosomal frameshifting of SARS-coronavirus by structure-based virtual screening. J. Am. Chem. Soc. 133, 10094–10100 (2011). 10.1021/ja1098325 21591761

[44] Y. Sun, L. Abriola, Y. V. Surovtseva, B. D. Lindenbach, J. U. Guo, Restriction of SARS-CoV-2 replication by targeting programmed −1 ribosomal frameshifting in vitro. bioRxiv 2020.10.21.349225 (2020). 10.1101/2020.10.21.349225 34185680 PMC8256030

[45] A. Sharma, M. Mariappan, S. Appathurai, R. S. Hegde, In vitro dissection of protein translocation into the mammalian endoplasmic reticulum. Methods Mol. Biol. 619, 339–363 (2010). 10.1007/978-1-60327-412-8_20 20419420 PMC3122127

[46] S. Q. Zheng, E. Palovcak, J. P. Armache, K. A. Verba, Y. Cheng, D. A. Agard, MotionCor2: Anisotropic correction of beam-induced motion for improved cryo-electron microscopy. Nat. Methods 14, 331–332 (2017). 10.1038/nmeth.4193 28250466 PMC5494038

[47] K. Zhang, Gctf: Real-time CTF determination and correction. J. Struct. Biol. 193, 1–12 (2016). 10.1016/j.jsb.2015.11.003 26592709 PMC4711343

[48] J. Zivanov, T. Nakane, B. O. Forsberg, D. Kimanius, W. J. H. Hagen, E. Lindahl, S. H. W. Scheres, New tools for automated high-resolution cryo-EM structure determination in RELION-3. eLife 7, e42166 (2018). 10.7554/eLife.42166 30412051 PMC6250425

[49] A. Punjani, J. L. Rubinstein, D. J. Fleet, M. A. Brubaker, cryoSPARC: Algorithms for rapid unsupervised cryo-EM structure determination. Nat. Methods 14, 290–296 (2017). 10.1038/nmeth.4169 28165473

[50] A. Punjani, D. J. Fleet, 3D variability analysis: Resolving continuous flexibility and discrete heterogeneity from single particle cryo-EM. J. Struct. Biol. 213, 107702 (2021). 10.1016/j.jsb.2021.107702 33582281

[51] T. C. Terwilliger, O. V. Sobolev, P. V. Afonine, P. D. Adams, Automated map sharpening by maximization of detail and connectivity. Acta Crystallogr. D Struct. Biol. 74, 545–559 (2018). 10.1107/S2059798318004655 29872005 PMC6096490

[52] S. Shao, J. Murray, A. Brown, J. Taunton, V. Ramakrishnan, R. S. Hegde, Decoding mammalian ribosome-mRNA states by translational GTPase complexes. Cell 167, 1229–1240.e15 (2016). 10.1016/j.cell.2016.10.046 27863242 PMC5119991

[53] E. F. Pettersen, T. D. Goddard, C. C. Huang, G. S. Couch, D. M. Greenblatt, E. C. Meng, T. E. Ferrin, UCSF Chimera—A visualization system for exploratory research and analysis. J. Comput. Chem. 25, 1605–1612 (2004). 10.1002/jcc.20084 15264254

[54] P. Emsley, B. Lohkamp, W. G. Scott, K. Cowtan, Features and development of Coot. Acta Crystallogr. D Biol. Crystallogr. 66, 486–501 (2010). 10.1107/S0907444910007493 20383002 PMC2852313

[55] D. Liebschner, P. V. Afonine, M. L. Baker, G. Bunkóczi, V. B. Chen, T. I. Croll, B. Hintze, L. W. Hung, S. Jain, A. J. McCoy, N. W. Moriarty, R. D. Oeffner, B. K. Poon, M. G. Prisant, R. J. Read, J. S. Richardson, D. C. Richardson, M. D. Sammito, O. V. Sobolev, D. H. Stockwell, T. C. Terwilliger, A. G. Urzhumtsev, L. L. Videau, C. J. Williams, P. D. Adams, Macromolecular structure determination using x-rays, neutrons and electrons: Recent developments in Phenix. Acta Crystallogr. D Struct. Biol. 75, 861–877 (2019). 10.1107/S2059798319011471 31588918 PMC6778852

[56] K. J. Webb, C. I. Zurita-Lopez, Q. Al-Hadid, A. Laganowsky, B. D. Young, R. S. Lipson, P. Souda, K. F. Faull, J. P. Whitelegge, S. G. Clarke, A novel 3-methylhistidine modification of yeast ribosomal protein Rpl3 is dependent upon the YIL110W methyltransferase. J. Biol. Chem. 285, 37598–37606 (2010). 10.1074/jbc.M110.170787 20864530 PMC2988365

[57] A. Brown, S. Shao, J. Murray, R. S. Hegde, V. Ramakrishnan, Structural basis for stop codon recognition in eukaryotes. Nature 524, 493–496 (2015). 10.1038/nature14896 26245381 PMC4591471

[58] N. W. Moriarty, P. D. Adams, Iron-sulfur clusters have no right angles. Acta Crystallogr. D Struct. Biol. 75, 16–20 (2019). 10.1107/S205979831801519X 30644841 PMC6333285

[59] P. P. Chan, T. M. Lowe, GtRNAdb 2.0: An expanded database of transfer RNA genes identified in complete and draft genomes. Nucleic Acids Res. 44, D184–D189 (2016). 10.1093/nar/gkv1309 26673694 PMC4702915

[60] N. K. Sinha, A. Ordureau, K. Best, J. A. Saba, B. Zinshteyn, E. Sundaramoorthy, A. Fulzele, D. M. Garshott, T. Denk, M. Thoms, J. A. Paulo, J. W. Harper, E. J. Bennett, R. Beckmann, R. Green, EDF1 coordinates cellular responses to ribosome collisions. eLife 9, e58828 (2020). 10.7554/eLife.58828 32744497 PMC7486125

[61] A. W. Schüttelkopf, D. M. F. van Aalten, PRODRG: A tool for high-throughput crystallography of protein-ligand complexes. Acta Crystallogr. D Biol. Crystallogr. 60, 1355–1363 (2004). 10.1107/S0907444904011679 15272157

[62] V. B. Chen, W. B. Arendall III, J. J. Headd, D. A. Keedy, R. M. Immormino, G. J. Kapral, L. W. Murray, J. S. Richardson, D. C. Richardson, MolProbity: All-atom structure validation for macromolecular crystallography. Acta Crystallogr. D Biol. Crystallogr. 66, 12–21 (2010). 10.1107/S0907444909042073 20057044 PMC2803126

[63] F. Sievers, A. Wilm, D. Dineen, T. J. Gibson, K. Karplus, W. Li, R. Lopez, H. McWilliam, M. Remmert, J. Söding, J. D. Thompson, D. G. Higgins, Fast, scalable generation of high-quality protein multiple sequence alignments using Clustal Omega. Mol. Syst. Biol. 7, 539 (2011). 10.1038/msb.2011.75 21988835 PMC3261699

[64] X. Robert, P. Gouet, Deciphering key features in protein structures with the new ENDscript server. Nucleic Acids Res. 42, W320–W324 (2014). 10.1093/nar/gku316 24753421 PMC4086106

[65] G. Loughran, M. T. Howard, A. E. Firth, J. F. Atkins, Avoidance of reporter assay distortions from fused dual reporters. RNA 23, 1285–1289 (2017). 10.1261/rna.061051.117 28442579 PMC5513072

[66] B. W. Dyer, F. A. Ferrer, D. K. Klinedinst, R. Rodriguez, A noncommercial dual luciferase enzyme assay system for reporter gene analysis. Anal. Biochem. 282, 158–161 (2000). 10.1006/abio.2000.4605 10860516

[67] N. J. McGlincy, N. T. Ingolia, Transcriptome-wide measurement of translation by ribosome profiling. Methods 126, 112–129 (2017). 10.1016/j.ymeth.2017.05.028 28579404 PMC5582988

[68] M. Martin, Cutadapt removes adapter sequences from high-throughput sequencing reads. EMBnet.journal 17, 10 (2011). 10.14806/ej.17.1.200

[69] T. Smith, A. Heger, I. Sudbery, UMI-tools: Modeling sequencing errors in Unique Molecular Identifiers to improve quantification accuracy. Genome Res. 27, 491–499 (2017). 10.1101/gr.209601.116 28100584 PMC5340976

[70] B. Langmead, S. L. Salzberg, Fast gapped-read alignment with Bowtie 2. Nat. Methods 9, 357–359 (2012). 10.1038/nmeth.1923 22388286 PMC3322381

[71] R. Edgar, M. Domrachev, A. E. Lash, Gene Expression Omnibus: NCBI gene expression and hybridization array data repository. Nucleic Acids Res. 30, 207–210 (2002). 10.1093/nar/30.1.207 11752295 PMC99122

[72] V. G. George, J. C. Hierholzer, E. W. Ades, “Cell culture” in *Virology Methods Manual*, B. W. J. Mahy, H. O. Kangro, Eds. (Academic Press, 1996), pp. 3–24.

[73] J. Schindelin, I. Arganda-Carreras, E. Frise, V. Kaynig, M. Longair, T. Pietzsch, S. Preibisch, C. Rueden, S. Saalfeld, B. Schmid, J. Y. Tinevez, D. J. White, V. Hartenstein, K. Eliceiri, P. Tomancak, A. Cardona, Fiji: An open-source platform for biological-image analysis. Nat. Methods 9, 676–682 (2012). 10.1038/nmeth.2019 22743772 PMC3855844

[74] J. Mutterer, E. Zinck, Quick-and-clean article figures with FigureJ. J. Microsc. 252, 89–91 (2013). 10.1111/jmi.12069 23906423

[75] T. D. Goddard, C. C. Huang, E. C. Meng, E. F. Pettersen, G. S. Couch, J. H. Morris, T. E. Ferrin, UCSF ChimeraX: Meeting modern challenges in visualization and analysis. Protein Sci. 27, 14–25 (2018). 10.1002/pro.3235 28710774 PMC5734306

[76] G. Cardone, J. B. Heymann, A. C. Steven, One number does not fit all: Mapping local variations in resolution in cryo-EM reconstructions. J. Struct. Biol. 184, 226–236 (2013). 10.1016/j.jsb.2013.08.002 23954653 PMC3837392

[77] V. Chandrasekaran, S. Juszkiewicz, J. Choi, J. D. Puglisi, A. Brown, S. Shao, V. Ramakrishnan, R. S. Hegde, Mechanism of ribosome stalling during translation of a poly(A) tail. Nat. Struct. Mol. Biol. 26, 1132–1140 (2019). 10.1038/s41594-019-0331-x 31768042 PMC6900289

[78] W. A. Cantara, P. F. Crain, J. Rozenski, J. A. McCloskey, K. A. Harris, X. Zhang, F. A. P. Vendeix, D. Fabris, P. F. Agris, The RNA modification database, RNAMDB: 2011 update. Nucleic Acids Res. 39, D195–D201 (2011). 10.1093/nar/gkq1028 21071406 PMC3013656

